# The Inflammatory Role of Platelets: Translational Insights from Experimental Studies of Autoimmune Disorders

**DOI:** 10.3390/ijms17101723

**Published:** 2016-10-14

**Authors:** Susann Pankratz, Stefan Bittner, Beate E. Kehrel, Harald F. Langer, Christoph Kleinschnitz, Sven G. Meuth, Kerstin Göbel

**Affiliations:** 1 Department of Neurology, University of Münster, 48149 Münster, Germany; sven.meuth@ukmuenster.de; 2 Department of Neurology, University Medical Center of the Johannes Gutenberg-University, 55131 Mainz, Germany; stefan.bittner@unimedizin-mainz.de; 3 Department of Anesthesiology, Intensive Care and Pain Medicine, Experimental and Clinical Hemostasis, University of Münster, 48149 Münster, Germany; haemostasis.research@uni-muenster.de; 4 University Clinic for Cardiology and Cardiovascular Medicine, Eberhard Karls-University Tübingen, 72076 Tübingen, Germany; Harald.Langer@med.uni-tuebingen.de; 5 Section for Cardioimmunology, Eberhard Karls-University Tübingen, 72076 Tübingen, Germany; 6 Department of Neurology, University Hospital Essen, 45147 Essen, Germany; Christoph.Kleinschnitz@uk-essen.de

**Keywords:** platelets, autoimmunity, neuroinflammation

## Abstract

Beyond their indispensable role in hemostasis, platelets have shown to affect the development of inflammatory disorders, as they have been epidemiologically and mechanistically linked to diseases featuring an inflammatory reaction in inflammatory diseases like multiple sclerosis, rheumatoid arthritis and inflammatory bowel disorders. The identification of novel molecular mechanisms linking inflammation and to platelets has highlighted them as new targets for therapeutic interventions. In particular, genetic and pharmacological studies have identified an important role for platelets in neuroinflammation. This review summarizes the main molecular links between platelets and inflammation, focusing on immune regulatory factors, receptors, cellular targets and signaling pathways by which they can amplify inflammatory reactions and that make them potential therapeutic targets.

## 1. Introduction

Platelets are tiny anucleate cells that circulate in a quiescent discoid state in the blood stream [1]. Their well-known physiological function is to regulate hemostasis as cellular effectors of hemostasis. In this context, platelets are rapidly deployed to sites of vascular injury, where they are indispensable for orchestration to stop blood loss [2]. They adhere to exposed subendothelial matrix proteins of the damaged vessel wall such as collagen, von Willebrand factor (vWF) or collagen bound fibrinogen [3]. Through this firm interaction with the endothelium, platelets become locally activated and consequently change their shape from discoid to pseudopodia state. This activation includes the release of different mediators from their storage compartments into circulation, which, in turn, can activate and recruit additional platelets to the endothelial lesion [4]. Crosslinking of adjacent activated platelets along with the binding of fibrinogen to glycoprotein (Gp) IIb/IIIa (also known as integrin α_IIb_β_3_ or cluster of differentiation (CD) 41/CD61) on activated platelets results in platelet aggregation [5]. The forming primary platelet plug can limit bleeding and provides sealing of the endothelial wound. However, it has been shown that platelet activation is not just linked to beneficial effects in hemostasis. Dysregulation of the coagulation cascade can lead to an aberrant activation and, thus, aggregation of platelets that is mostly associated with cardiovascular pathogenesis (reviewed in detail elsewhere [6,7,8,9]). In addition to their well-understood and indispensable hemostatic role, platelets are receiving more and more interest as immune and inflammatory effector cells [10]. There is growing evidence that they actively participate in various immune-mediated pathogenic circumstances [11].

In this review, we summarize key links between platelets and inflammation, with a specific focus on molecular pathways in autoinflammatory disorders. The evidence presented here suggests that manipulation of platelets could be potentially therapeutically exploitable in autoimmune diseases in general.

## 2. Platelets—Cellular Mediators of (Neuro-) Inflammation

Given the fact that platelets are not only cellular effectors of hemostasis, but actively assist immune mediated (neuro-) inflammation, it is not surprising that platelets secret and shed a range of mediators both relevant for hemostasis and the immune response [12].

Platelets usually circulate in a quiescent state in the blood circulation, where they contact a variety of substances that activate them, including lipopolysaccharides (LPS) and toll-like receptor (TLR) ligands along with thrombin, collagen and adenosine diphosphate (ADP) [13,14]. Upon activation, platelets can directly and indirectly communicate with several target cells (e.g., leukocytes, endothelial cells) that are involved in the initiation and propagation of (neuro-) inflammatory reactions through various platelet-derived factors. These various factors can thus mediate activation, recruitment and transmigration of involved target cells. These platelet-derived factors can include the release of cytokines, chemokines and other mediators (like serotonin and ADP) from their main storage compartments (α- and dense granules). Further, current studies indicate that platelet activation can also lead to de novo synthesis of mediators, like cytokines, by their regulated metabolic activity [15,16]. Beyond to their capacity to store and secrete immune modulatory molecules, activated platelets are highly effective at generating extracellular vesicles named microparticles (MP). Despite the fact that MP can be of various cellular origin (e.g., endothelial cells), platelet MP (PMP) represent the primary source of MP in the blood circulation [17]. The PMP content harbor an elaborate set of transcription factors, enzymes, micro ribonucleic acid and various mediators that can be delivered to surrounding target cells to impact their function [16]. Moreover, PMP, like platelets, expose a range of surface proteins which enable them both to provide binding sites for adjacent target cells (e.g., leukocytes, endothelial cells) and to deliver surrounded stimuli leading to the secretion of cytokines/chemokines, under the control of specific intracellular regulatory pathways. The main platelet receptors, which comprise TLR, siglecs, Gp as well as metabotropic purinergic receptors, and their ligands are reviewed in detail elsewhere [14,18,19,20].

Increased amounts of platelet-derived factors along with increased activation status of platelets occur in the pathogenesis of several immune mediated inflammatory diseases.

## 3. Role of Platelet-Driven Neuroinflammation in Multiple Sclerosis

Multiple sclerosis (MS) is an inflammatory disease of the central nervous system (CNS), characterized by demyelination of neuronal axons. Although the aetiology and pathogenesis of MS are still not completely understood, it is widely accepted that MS is an immune-mediated disease [21]. It can be assumed that immunological processes during the initial phases of disease include the formation of self-reactive leukocytes in the peripheral circulation that eventually transmigrate across the activated blood-brain barrier (BBB). The subsequent disruption of the impermeable nature of the BBB facilitates the local recruitment of further inflammatory effector cells into the CNS parenchyma mediating tissue damage.

**Figure 1:**
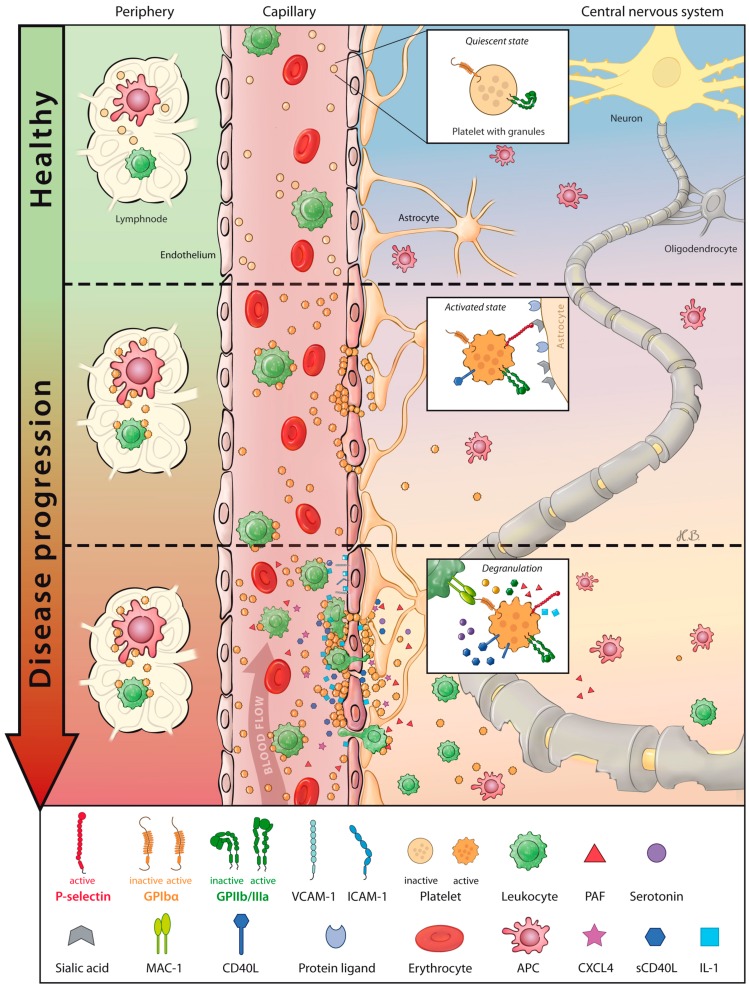
Platelet participation in pathophysiology of multiple sclerosis.

Emerging new concepts emphasize that factors that do not belong to the immune system are involved in inflammatory degeneration in MS. In particular, constituents of the plasmatic coagulation system and the contact systems have received interest [22,23]. Further, depositions of coagulation factors, such as fibrinogen or factor XII, were described in plaques of MS patients [23,24,25]. Besides the plasmatic coagulation, slight attention has also been given to platelets in the pathogenesis of MS in the past, despite the fact that early evidence from 1950s/60s pointed to platelet abnormalities in MS patients [26,27,28,29]. These data already suggested an immune mediated role for platelet contribution in MS, but were then largely forgotten in future generations of MS researchers. Indeed, current studies suggest that platelet depletion reduces disease severity and inflammation in mice that were subjected to experimental autoimmune encephalomyelitis (EAE), a classical animal model of CNS inflammation to study the pathogenesis of MS [30,31,32]. In particular, a number of studies have demonstrated that platelets are present along with an increased activation status in the peripheral blood and in plaques of MS patients as indicated by elevated PMP levels, P-selectin expression (also known as CD62P), increased levels of platelet-activating factor (PAF) and upregulation of GpIIb receptor (see Figure 1) [30,33,34,35,36]. Of note, the PMP level was higher in untreated MS patients and relapsing-remitting patients showed the highest levels both compared to respective controls, thus emphasizing a predictive role as biomarker [35]. Consistent with the findings in MS patients, an increased activation status of platelets has also found in inflamed CNS tissue of EAE mice [30,31,37], whereby EAE symptoms correlate with the levels of PAF [38]. Additionally, the genetic ablation of PAF receptor in EAE (see Table 1) led to decreased disease severity along with reduced CNS inflammation and demyelination [38]. Elevated platelet activation was linked to the interaction between platelets and CNS-specific cell compartments. Thereby, it was shown that platelets have the ability to recognize gangliosides (sialic acids) within the lipid rafts on the surface of astrocytes and neurons due to the disrupted integrity of the BBB. The relevance of platelet recognition of CNS-specific glycolipid structures is underscored by animal studies with reinforcement/inhibition and genetic ablation of platelet glycolipid interaction (see Table 1 and Table 2). Platelet activation (expression of P-selectin) was triggered through this interaction following secretion of pro-inflammatory mediators, interleukin (IL)-1, platelet factor (PF)-4, and 5-hydroxytryptamine (5-HT or serotonin, see Figure 1) [31]. Interestingly, platelet-derived serotonin possesses the ability to recruit leukocytes (neutrophils) to the site of CNS inflammation [39]. Another experimental evidence revealed that 5-HT transporter knockout (5-HTT^−/−^, see Table 1) mice showed a decreased disease severity along with reduced CNS inflammation in EAE [40]. In line with this, treatment of relapsing MS patients with the antidepressant fluoxetine, which is a selective serotonin-reuptake inhibitor, reduced the disease activity due to its neuroprotective effect [41,42].

Full platelet activation can be induced in response to platelet-endothelial cell interactions. Note that, under physiological conditions, circulating platelets do not interact with intact endothelium. However, it is reported that platelets can also alter the phenotype of endothelial cells. In this context, it was demonstrated that platelets can indirectly contribute to the activation of cerebrovascular endothelium via released IL-1α. In addition, endothelial activation in response to platelet-derived IL-1α was associated with the expression of cell adhesion molecules, intracellular adhesion molecule-1 (ICAM-1) and vascular cell adhesion protein (VCAM-1) as well as to enhanced release of CXC chemokine ligand 1 (CXCL1) [43]. Studies with human endothelial cells revealed similar results [44]. Besides IL-1, platelets can also modulate the BBB via their expression of CD40 Ligand (CD40L, also known as CD154). Like IL-1, CD40L enable platelets to indirectly interact with endothelium, which express CD40, thus triggering upregulation of adhesion molecules and chemokine [45]. The contribution of E- and P-selectin, additional adhesion molecules, in EAE pathogenesis were investigated (see Table 1), thus showing that they are not required for the development of EAE although P-selectin was found to be upregulated on both endothelium and platelets in inflamed CNS [37]. Nevertheless, the genetic ablation of platelet/endothelial cell adhesion molecule-1 (PECAM-1, also known as CD31) in animal studies of EAE (see Table 1) revealed that PECAM-1 plays a major role in the restoration of endothelial integrity [46].

As already mentioned, activation of platelets can be modulated by distinct receptors. In this context, the metabotropic purinergic ADP receptor P2Y_12_, which is mainly found on platelets, seems to play a significant role. Experimental inhibition of P2Y_12_ receptor by clopidogrel on human platelets showed a reduced release of both P-selectin and CD40 Ligand (CD40L) [47]. The relevance of P2Y_12_ receptor inhibitors like clopidogrel or prasugrel in MS patients has not yet been analyzed, but platelets can bind to CNS-specific lipid rafts through CD62P [31] and CD40L triggers endothelial activation [45], meaning both mediators fire neuroinflammatory processes. Furthermore, it was shown that dipyridamole decreased the clinical severity of EAE (see Table 2), although other molecular mechanisms than inhibition of platelet activation were relevant in this context [48]. Blockade of platelet key receptors (see Table 2) showed that platelets participate in EAE pathogenesis by recognizing integrins [30]. Here, it should be taken into account that Gp blocker might be an opportunity to treat MS. Copaxone, which are already successfully applied for MS therapy, seem to beneficially affect platelet activation additional to its known mode of action [49].

Although an increasing body of evidence clearly shows the inflammatory role of platelets and their derived factors/receptors in pathophysiology of MS and EAE, the role of platelets remain elusive so far. For instance, it is known that disease activity in MS undergoes shifts in the time before, during and after pregnancy with a clear reduction in relapse rates, especially in the last trimester, of 70%–80% [50]. Especially, in late pregnancy, it is known that increased platelet activation occurs and is associated with increased concentrations of β-thromboglobulin and thromboxane A2 [51,52]. However, whether these findings hold also true in MS patients has not been examined so far, so further studies are required.

## 4. Role of Platelet-Driven Immune Responses in Other Non-Neurological Inflammatory Disorders

An increasing body of evidence also supports the role of platelets and platelet-derived factors in non-neurological auto-inflammatory diseases. In the following, we focus on two well-characterized disorders (rheumatoid arthritis (RA) and inflammatory bowel disorders (IBD)) to elaborate the immunological role of platelets.

## 5. Rheumatoid Arthritis

In particular, a substantial contribution of platelet-derived factors has been suggested in rheumatoid arthritis (RA; reviewed in detail elsewhere [53]) as platelet glycoprotein IIb/IIIa can be detected in the synovium of patients with RA [54,55]. Moreover, the activation marker P-selectin is higher in platelets from patients with active RA than those in remission [56] and elevated levels of PMP and soluble CD40L as well as P-selectin are reported in individuals suffering from RA [57,58,59,60,61,62,63]. Further, platelet aggregates and platelet or PMP adherent to leukocytes have been detected in both blood and synovial fluids of RA patients [58,64,65,66,67,68,69,70]. In vitro, it was shown that platelets are hyper-responsive to further activation, potentially reflecting the in vivo priming of platelets during disease [71,72].

A further direct pro-inflammatory role of platelets was suggested as its depletion in mice leads to an improvement in the clinical symptoms in an animal model of RA and results in decreased inflammation, bone and cartilage erosion (see Table 3) [70]. Interestingly, it was shown that GPVI is the relevant receptor for this effect, while neither thromboxane production or blockade of its receptor nor GPIbα was necessary in this context (see Table 3 and Table 4) [70]. Furthermore, it was shown that genetic depletion of COX-1 in mice resulted in an ameliorated disease course in an animal model of RA [53]. Even more surprising, treatment with P2Y_12_ receptor antagonists like prasugrel leads to an aggravated disease course [70,73]; however, to support the potential human relevance, several case reports of patients developing spontaneous joint inflammation after clopidogrel intake exist [74,75,76,77,78,79]. Additionally, the effect of dipyridamole was tested as a therapy in RA patients, but it did not modify disease severity [80].

Thus, platelet activation mechanisms engaged during RA seems to be different from the typical pathways involved in thrombosis, so that not all drugs currently used to inhibit platelet function might have a protective effect in the context of arthritis.

### Inflammatory Bowel Disease

In addition to RA, potential contribution of platelets and platelet-derived factors is also under consideration for inflammatory bowel disease (IBD), namely Crohn’s disease and ulcerative colitis (reviewed in detail elsewhere [87,88]). These disorders are known to be associated with an increased risk for thromboembolism [87]. Interestingly, there is evidence that patients suffering from IBD reveal a larger number but smaller size of platelets. This finding is proposed to be used as a marker for disease activity [89,90,91,92,93]. Furthermore, it was shown that both expression of P-selectin, GP53 and CD40L on circulating platelets, appearance of platelet-leukocyte aggregates and soluble CD40L, β-thromboglobulin and PF-4 are increased in IBD patients [94,95,96,97]. In line with these findings, animal models of colitis have demonstrated accumulation of platelets in colonic venules that correlated with disease activity and adherent leukocytes and were predominantly attached to the surface of leukocytes [81,82]. In this model, pharmacological depletion of platelets induces reduced rolling and adhesion of leukocytes [81]. Genetic depletion or pharmacological blockade of both P-selectin of PSGL-1 led to reduced platelet and leukocyte adhesion in mice, while findings concerning disease activity revealed contradictory results (see Table 3 and Table 4) [81,82,83,84,85,86]. Furthermore, the same group could show that genetic depletion of CD40 or its ligand is protective in animal model of colitis with reduced platelet and leukocyte adhesion (see Table 3). Nonetheless, blockade of GPIIb/IIIa had no effect on platelet adhesion in an experimental model of colitis [82]. In contrast to RA, pharmacological blockade of P2Y_12_ receptor by clopidogrel or treatment with acetylsalicylic acid reveals an attenuated disease course in models of IBD [98,99].

Collectively, these results demonstrate a clear role of platelets and platelet-derived factors, not only in neuroinflammation, but also in other autoimmune disorders.

## 6. Future Prospects/Concluding Remarks

Beyond their hemostatic functions, platelets have the capacity to produce and secrete a variety of immune modulatory mediators after activation in response to various factors leading to initiation and modulation of immune-mediated inflammatory processes via interaction with other platelets, leukocytes and the endothelium. In this context, increased amounts of platelet-derived factors along with an increased activation status of platelets occur in the pathogenesis of MS, RA and IBD, emphasizing that modulating platelet activity may be beneficial. Furthermore, platelets and platelet-associated molecules have been tested as targets for imaging tools in vascular inflammation [100]. Thereby, platelets and their activation status merit consideration as a tool and biomarker in immune-mediated disease prediction [35] and also as targets for therapy of (neuro-)inflammation [49,101]. A recent report has implicated platelets as effectors of tissue remodeling processes such as apoptosis after brain damage using a model of transient ischemia [102]. This unexpected role of platelets within the neuronal tissue will have to be further questioned in additional experimental settings. In conclusion, further research is still required to elucidate the underlying mechanism(s) by which platelets, their receptors and platelet-derived factors modulate vascular integrity, and leukocyte crosstalk will aid in our understanding of the role platelets might play during inflammatory immune responses. Some of these targets already have available drugs, which creates an opportunity to merely repurpose them for use in immune mediated disease, like MS.

Under steady state, platelets circulate in a quiescent state in the blood peripheral blood stream. Disruption of the impermeable nature of the BBB is a pathophysiological hallmark of MS and its animal model, EAE. Platelets rapidly deployed to sites of neurovascular injury to restore endothelial integrity and interact with lipid rafts within CNS parenchyma. This initial interaction is possible since platelets recognize sialic acids within the lipid rafts, which results in the expose of P-selectin on platelet surface. Upon activation, platelets active secret chemokines/cytokines and upregulate surface molecules, which both enable them to more efficiently communicate with target cells. Thereby, platelets mediate leukocyte recruitment and activation of endothelial cells. Leukocytes adhere to activated endothelium through adhesion molecules that promote leukocyte–endothelial interaction and leukocyte transmigration. Thus, platelets facilitate immune cell infiltration from the periphery into the CNS, where they may become reactivated and fan the fire of neuroinflammation.

## Figures and Tables

**Table 1:** Studies of platelets, their receptors and molecules: effects on inflammatory processes of the central nervous system in transgenic mice.

Mouse Line (Genetic Background)	Model (Peptide)	Inflammatory Effect	Referene
*ST3Gal-V^−^*^/*−*^ (C57BL/6)	EAE (MOG_35–55_)	Ameliorated disease course due to lack of brain-specific gangliosides that can recognize by platelets. Reduced CNS inflammation as determined by the infiltration of less lymphocytes, CD4 T cells and macrophages on day 21 after the EAE induction.	[31]
*E-/P-selectin^−^*^/*−*^ (C57BL/6)	EAE (MOG_35–55_)	No effect on clinical symptoms.	[37]
*E-/P-selectin^−/−^* (SJL)	EAE (PLP_139–151_)	No effect on clinical symptoms.	[37]
*5-HTT^−/−^*(C57BL/6)	EAE (MOG_35–55_)	Decreased disease severity. Reduced CNS inflammation.	[40]
*5-HTT^−/−^* (C57BL/6)	EAE (rat MBP)	Decreased disease severity.	[40]
*PAF receptor^−/−^* (C57BL/6)	EAE (MOG_35–55_)	Decreased disease severity. Reduced CNS inflammation and demyelination.	[38]
*FcR γ-chain ^−/−^* (C57BL/6J)	EAE (MOG_35–55_)	Reduced clinical symptoms.	[36]
*PECAM-1^−/−^* (C57BL/6)	EAE (MOG_35–55_)	Early onset of clinical symptoms associated with early leukocyte migration into CNS.	[46]
*PECAM-1^−/−^* (C57BL/6)	Adoptive transfer of EAE (MOG_35–55_)	Early onset of clinical symptoms regardless of whether KO mice were injected with MOG_35–55_-specific WT or PECAM-1^−/−^ T cells.	[46]

Abbreviations: CD, cluster of differentiation; CNS, central nervous systems; EAE, experimental autoimmune encephalomyelitis; FcR γ-chain, Fc receptor γ-chain; Gp, glycoprotein; 5-HTT, 5-hydroxytryptamine (common name serotonin) transporter; IL, interleukin; KO, knockout; MBP, myelin basic protein; MOG_35–55_, myelin oligodendrocyte glycoprotein 35–55; PAF, platelet-activating factor; PECAM-1, platelet/endothelial cell adhesion molecule-1 (also known as CD31); PLP_139–151_, proteolipid protein 139–151; WT, wild type.

**Table 2:** Studies of platelets, their receptors and molecules contributing to neurovascular inflammation using pharmacological substances/experimental manipulations.

Treatment	Model (Peptide)	Genetic Background/or Species	Inflammatory Effect	Reference(s)
Injection of brain lipid rafts on day 0 (platelet degranulation within brain)	EAE (MOG_35–55_) without PTx	C57BL/6	EAE was induced.	[31]
Intracranial injection of platelet rich plasma on day 0 (systemic platelet degranulation)	EAE (MOG_35–55_) without PTx	C57BL/6	EAE was induced.	[31]
Neuroaminidase (Prevention of platelet-lipid rafts interactions)	EAE (MOG_35–55_)	C57BL/6	Decreased disease severity.	[31]
LFA protein (Prevention of platelet-lipid rafts interactions)	EAE (MOG_35–55_)	C57BL/6	Decreased disease severity.	[31]
CTB (Prevention of platelet-lipid rafts interactions)	EAE (MOG_35–55_)	C57BL/6	Decreased disease severity.	[31]
Anti-GQ Ab (Prevention of platelet-lipid rafts interactions)	EAE (MOG_35–55_)	C57BL/6	Decreased disease severity.	[31]
Anti-M2 Ab on days 12, 14 & 16 (blockig Mac-1/GP1bα interaction)	EAE (MOG_35–55_)	C57BL/6	Decreased disease severity.	[30]
Anti-GPIIb/IIIα Fab on days 12, 14 & 16	EAE (MOG_35–55_)	C57BL/6	Decreased disease severity.	[30]
Anti-GPIbα Fab on days 12, 14 & 16	EAE (MOG_35–55_)	C57BL/6	Decreased disease severity.	[30]
Anti-GPIbα Fab on days 15, 17 & 19	EAE (MOG_35–55_)	C57BL/6	Decreased disease severity.	[30]
Anti-thrombocyte serum (platelet depletion) on days 0, 2, 4 & 8 or 12 & 16	EAE (MOG_35–55_)	C57BL/6	Decreased disease severity. Reduced CNS inflammation.	[30,31]
Anti- thrombocyte serum (platelet depletion) on days 2 & 6	EAE (MOG_35–55_)	C57BL/6	No effect on clinical symptoms.	[30]
Dipyridamole	EAE (MOG_35–55_)	C57BL/6	Reduced clinical symptoms, decerased microglial activity.	[48]

Abbreviations: Ab, antibody; CD, cluster of differentiation; CNS, central nervous systems; CTB, β subunit of cholera toxin; EAE, experimental autoimmune encephalomyelitis; Fab, Fragment antigen binding; FcR γ-chain, Fc receptor γ-chain; Gp, glycoprotein; LFA, Limax flavus agglutinin; MOG_35–55_, myelin oligodendrocyte glycoprotein 35–55; PECAM-1, platelet/endothelial cell adhesion molecule-1 (also known as CD31); PF4, platelet factor 4 (also known as CXCL4); PLP_139–151_, proteolipid protein 139–151; PTx, Pertussis toxin; WT, wildtype.

**Table 3:** Studies of platelets and platelet-derived factors: effects on inflammatory processes in transgenic mice.

Mouse Line (Genetic Background)	Model (Peptide)	Inflammatory Effect	Reference
*Cox1^−/−^*	K/BxN serum transfer arthritis (K/BxN serum)	Ameliorated disease course due to platelet-derived COX-1	[53]
*Gp1ba^−/−^* (C57BL/6)	K/BxN serum transfer arthritis (K/BxN serum)	No clinical effect	[70]
*Gp6^−/−^* (C57BL/6)	K/BxN serum transfer arthritis (K/BxN serum)	Decreased disease severity. Reduced inflammation, bone erosion, cartilage erosion	[70]
*Tbxas1^−/−^* (C57BL/6)	K/BxN serum transfer arthritis (K/BxN serum)	No effect on clinical symptoms	[70]
*CD40^−/−^* (C57BL/6)	Colitis (DSS)	Attenuated disease activity, reduced inflammation and MPO activity, reduced platelet and leukocyte adhesion	[81]
*CD40L^−/−^* (C57BL/6)	Colitis (DSS)	Attenuated disease activity, reduced inflammation and MPO activity, reduced platelet and leukocyte adhesion	[81]
*P-Selectin^−/−^* (C57BL/6)	Colitis (DSS)	Reduced platelet adhesion and rolling	[81]
		Reduced platelet adhesion, decreased albumin extravasation	[82]
		Enhanced disease activity, reduced MPO activity, inflammation, leukocyte rolling	[83]
*PSGL-1^−/−^* (C57BL/6)	Colitis (DSS)	Reduced platelet adhesion and rolling	[81]
		Earlier disease onset, enhanced disease activity, enhanced infiltration, platelets not examined	[84]
		Decreased disease activity, inflammation, reduced Th1 and Th17 infiltration, platelets not examined	[85]
*PSGL-1^−/−^* (Balb/c)	Colitis (DSS)	Reduced clinical disease activity, decreased leukocyte rolling, inflammation and MPO activity, platelets not examined	[86]

Abbreviations: Cox1, cyclooxygenase-1; DSS, dextran sulfate sodium ; Gp, glycoprotein; K/BxN, KRN and MHC class II molecule I-A (g7); MPO, myeloperoxidase; Tbxas1, Thromboxane A synthase 1.

**Table 4:** Studies of platelets and platelet-derived factors: effects on inflammatory processes using pharmacological substances.

Treatment	Model (Peptide)	Genetic Background/or Species	Inflammatory Effect	Reference
Anti-GPIbα Ab (platelet depletion)	K/BxN serum transfer arthritis (K/BxN serum)	C57BL/6J	Reduced clinical symptoms, decreased inflammation, bone erosion and cartilage erosion	[70]
Anti-GPIIb/IIa Ab	Colitis (DSS)	C57BL/6	No effect on adhesion of platelets and leukocytes	[82]
Anti-platelet serum	Colitis (DSS)	C57BL/6	Decreased rolling and adhesion of leukocytes	[81]
	Reactive arthritis (PG-PS)	Lewis rat	Exacerbated disease severity (increased joint diameter), increased synoviocyte hyperplasia (in both acute and chronic phases), blood vessel proliferation (chronic), inflammatory infiltration (acute and chronic) and fibrosis (chronic), increased IFNy, IL-1b, IL-6 plasma levels, reduced IL-10 plasma level, increased neutrophil and platelet count	[73]
	Crohn’s disease (TNBS)	Spargue Dawley rats	Attenuated disease course, reduced inflammation and MPO activity	[98]
	Ulcerative colitis (oxazolone)	Wistar rats	Attenuated disease course, reduced inflammation and MPO activity	[98]
Prasugrel (P2Y_12_ receptor antagonist)	Reactive arthritis (PG-PS)	Lewis rat	Exacerbated disease severity (increased joint diameter), greater synoviocyte hyperplasia, leukocyte infiltration, fibrosis, bone destruction, and pannus formation, increased platelet and neutrophil count, decreased IL-10 plasma levels	[73]
SQ29548 (Thromboxane A2 receptor antagonist)	K/BxN serum transfer arthritis (K/BxN serum)	C57BL/6J	No effect on clinical symptoms	[70]
Anti-P-Selectin Ab	Colitis (DSS)	C57BL/6	Decreased rolling and adhesion of platelets	[81]
			Reduced adhesion of platelets and leukocytes	[82]
			Reduced body weight loss, decreased disease activity, MPO activity, inflammation, leukocyte rolling in colon, enhanced MPO activity in lung	[83]
Anti-PSGL-1 Ab	Colitis (DSS)	C57BL/6	Decreased rolling and adhesion of platelets	[81]
			Reduced adhesion of platelets and leukocytes	[82]
Acetylsalicylic acid	Colitis (DSS)	C57BL/6	Decrease in disease severity	[99]
Clopidogrel	Colitis (oxazolone)	Rats	Decrease in disease severity, protection against mucosal damage	[98]
	Crohn’s disease (TNBS)	Rats	Decrease in disease severity, protection against mucosal damage	[98]

Abbreviations: Ab, antibody; AIA, antigen-induced arthritis; Gp, glycoprotein; IFNy, Interferon gamma; IL, Interleukin; K/BxN, KRN and MHC class II molecule I-A (g7); PG-PS, Peptidoglycan polysaccharide; TNBS, Trinitrobenzenesulphonic acid.

## Abbreviations

APC, antigen presenting cells; CD40L, cluster of differentiation 40 ligand; CXCL1, CXC-chemokine ligand 1; Gp, glycoprotein; ICAM-1, intracellular adhesion molecule-1; IL-1α, interleukin-1α; MAC-1; macrophage-1 antigen (also known as integrin αMβ2); sCD40L, soluble CD40 ligand; VCAM-1, Vascular cell adhesion protein 1.
